# Effects of straw return combined with different amendments on soil enzyme activities and bacterial community structure in acidic purple soil

**DOI:** 10.3389/fmicb.2026.1969469

**Published:** 2026-09-28

**Authors:** Binbin Liu, Hua Deng, Qiang Xu, Tianjie Han, Liyuan Mu

**Affiliations:** 1 Chongqing Youth Vocational & Technical College, Chongqing, China; 2 College of Resources and Environment, Southwest University, Chongqing, China

**Keywords:** acidic purple soil, bacterial community, soil health, soil management, sustainable agriculture

## Abstract

Soil microorganisms are fundamental drivers of nutrient cycling and ecosystem functioning in agricultural soils, but their responses to management practices in acidic agroecosystems remain insufficiently understood. Acidification-induced changes in soil environmental conditions can constrain bacterial activity and disrupt carbon and nutrient transformation processes. This study investigated how straw return combined with different amendments affects soil enzyme activities and bacterial community structure in acidic purple soil under a 2-year rice–rapeseed rotation system. Six management treatments, including control (CK), conventional fertilization (F), straw return (FT), and straw return combined with calcium–magnesium phosphate fertilizer (FGT), limestone powder (FST), or biochar (FBT), were established to evaluate their effects on soil properties, enzyme activities, and bacterial communities. The results showed that amendment treatments were associated with contrasting changes in soil biochemical properties and bacterial community characteristics. FST treatment effectively alleviated soil acidity and enhanced urease (S-UE) and peroxidase (S-POD) activities, whereas FBT treatment promoted soil organic carbon (SOC) accumulation and stimulated carbon-related enzyme activities. Amendment treatments were associated with variation in bacterial community composition, with treatment-specific differences in dominant bacterial taxa and their associations with soil environmental conditions. Soil pH, SOC, and enzyme activities were important environmental factors associated with bacterial community differentiation. These findings demonstrate that straw return combined with amendments regulates nutrient cycling through amendment-specific bacterial pathways, highlighting the importance of bacterial processes in restoring the health of acidic purple soils and improving the sustainability of their agricultural ecosystems in the Sichuan Basin.

## 1 Introduction

Soil microorganisms are central regulators of biogeochemical cycling in agroecosystems, controlling the transformation and availability of carbon, nitrogen, phosphorus, and other essential nutrients through diverse metabolic processes (Xiao et al., 2022). Microbial-mediated nutrient cycling directly influences soil fertility, crop productivity, and ecosystem sustainability. However, agricultural intensification has substantially altered soil microbial habitats, leading to disruptions in microbial diversity, functional activity, and nutrient transformation processes. Soil acidification represents an important form of agricultural soil degradation. It imposes dual adverse effects: on one hand, it limits agricultural productivity and regional ecological sustainability (Qasim et al., 2026), impairs soil fertility and crop nutrient uptake, and induces toxic Al activation and base cation leaching under reduced pH, raising nutrient loss risks to aquatic environments (Lu et al., 2025); on the other hand, it reshapes microbial communities and metabolism by altering habitats, disturbing organic matter decomposition, nutrient cycling and soil ecological processes (Meng et al., 2019). Previous studies have demonstrated that soil pH and soil organic carbon (SOC) are among the most important environmental drivers regulating microbial community succession and nutrient cycling processes (Rousk et al., 2009). Soil enzymes are important biochemical mediators of organic matter decomposition and nutrient transformation, and their activities can provide useful indicators of microbial nutrient acquisition and the potential turnover of soil C and N (Burns et al., 2013; Li et al., 2024a). Different enzymes reflect distinct biochemical processes: urease (S-UE) is associated with urea hydrolysis and N transformation, acid protease (S-ACPT) contributes to the decomposition of proteinaceous organic N, β-glucosidase (S-GC) is involved in the degradation of cellulose-derived C substrates, and peroxidase (S-POD) participates in the oxidative transformation of complex organic matter (Rahmanian and Khadem, 2024). Changes in these enzyme activities may therefore indicate how amendment-induced changes in soil pH and carbon availability affect microbial biochemical functioning. Against this background, identifying effective management strategies that simultaneously alleviate soil acidification, enhance SOC accumulation, and improve microbial functioning is essential for promoting soil quality in acidic purple soils.

As an important agricultural soil resource in the Sichuan Basin featuring rich mineral nutrients and high nutrient supply capacity, purple soil supports grain and oil production in the upper Yangtze River region (Zhu and Zhu, 2015). Long-term intensive cultivation, excessive fertilization and atmospheric acid deposition have accelerated its acidification, triggering nutrient imbalance, suppressed microbial activity and reduced crop productivity and threatening regional agricultural sustainability (Li et al., 2021). Therefore, clarifying the responses of acidic purple soils to different amendment strategies is important for improving soil quality and crop production in the region.

Straw return is widely acknowledged as an effective strategy for agricultural waste recycling and soil quality enhancement. By augmenting external organic carbon inputs, promoting soil aggregate formation, and stimulating microbial activity, it can improve soil fertility and ecological functions (He et al., 2025; Zhang et al., 2025). However, its efficacy is often constrained in acidic soils. On one hand, the release of low-molecular-weight organic acids during straw decomposition may further lower soil pH and exacerbate acidification; on the other hand, acidic conditions can suppress the activity of straw-degrading microorganisms, thereby reducing the efficiency of organic carbon transformation (Zhang et al., 2025). The application of soil amendments can effectively overcome the limitations of straw return alone. Lime, biochar, and calcium–magnesium phosphate fertilizer have been widely shown to improve the physicochemical properties of acidic soils, regulate nutrient cycling, and promote a more favorable soil microenvironment (Chen et al., 2026; Jiang et al., 2026). Lime rapidly neutralizes soil acidity, increases soil pH, and supplies exchangeable calcium, making it particularly effective for alleviating soil acidification (Li et al., 2026). Biochar, characterized by a highly porous structure and abundant surface functional groups, can increase soil organic carbon stocks, improve soil structure, and provide favorable microsites for microbial growth and activity (Liu et al., 2025). In contrast, calcium–magnesium phosphate fertilizer is a slow-release, multifunctional fertilizer that continuously supplies calcium, magnesium, and phosphorus, thereby reducing nutrient losses in acidic soils while regulating nutrient cycling and shaping microbial community structure to improve soil fertility and productivity (Yang et al., 2023). Despite their common role in soil improvement, these amendments operate through distinct pathways. Lime primarily alleviates soil acidification through rapid pH regulation and calcium supplementation, whereas calcium–magnesium phosphate fertilizer combines nutrient supply with gradual pH regulation and fertility enhancement. Biochar, in comparison, exerts a more persistent effect by increasing carbon inputs and sequestration while modifying the soil habitat and associated microbial processes. Thus, combining straw return with these functionally distinct amendments may generate complementary and synergistic effects, simultaneously improving soil chemical properties, enhancing carbon sequestration, and regulating microbial-mediated ecological processes.

In recent years, increasing attention has been devoted to the combined application of straw return and amendments. Previous studies have shown that straw return combined with lime application can increase soil pH in acidic paddy soils, enhance extracellular enzyme activities, optimize bacterial community composition, and promote carbon cycling-related functions (Zhang et al., 2026). Moreover, the synergistic application of straw and amendments has been reported to improve soil nutrient availability, alleviate acidification stress, stabilize crop productivity, and reduce the bioavailability of potentially toxic metals (Wang et al., 2026; Xu et al., 2026). Although previous studies have focused on the effects of straw return and amendments on soil fertility improvement, comparative evidence linking these amendment-specific changes in soil properties and enzyme activities with bacterial community responses in acidic purple soil remains limited. In particular, it remains unclear whether changes in soil pH and carbon availability are accompanied by consistent responses in soil enzyme activities and bacterial community composition under different amendment strategies.

To address these gaps, we conducted a two-year field experiment under a rice–rapeseed rotation in acidic purple soil in the Sichuan Basin. We compared straw return combined with limestone powder (FST), calcium–magnesium phosphate fertilizer (FGT), and biochar (FBT), with conventional fertilization and no-amendment treatments as references. We evaluated treatment effects on soil pH and SOC, four soil enzyme activities related to C and N transformation, bacterial diversity and community composition, and the relationships between bacterial communities and soil environmental variables. Based on the contrasting functions of the three amendments, we hypothesized that: (1) the three amendment strategies would produce distinct changes in soil pH, SOC, and enzyme activities; (2) these amendment-induced changes in soil conditions would be associated with differences in bacterial diversity and community composition, with different amendment strategies showing distinct taxon-specific responses; and (3) soil pH, SOC, and enzyme activities would be among the major environmental variables associated with bacterial community variation.

## 2 Materials and methods

### 2.1 Experimental site

The field experiment was conducted at the Jiangxiaobai Golden Huangzhuang Agricultural Park in Ciyun Town, Jiangjin District, Chongqing, China (106°12′47″, 29°06′30″). The study area is characterized by a subtropical monsoon climate, with an annual mean temperature of 17.9 °C, average annual precipitation of 1,034 mm, and an elevation of 270 m above sea level. The initial soil pH was 4.9, indicating a strongly acidic soil condition. The SOC content was 18.07 g/kg. The tested soil was classified as gray-brown purple soil, developed from the parent material of the Shaximiao Formation.

### 2.2 Test material

The rapeseed and rice cultivars used in this study were Qingyou 3 and Youyuzhen, respectively, both of which are well adapted to the local rice-rapeseed rotation system. The nitrogen, phosphorus, and potassium fertilizers applied in the experiment were urea (46% N), calcium superphosphate (12% P_2_O_5_), and potassium chloride (52% K_2_O), respectively. Three soil amendments were used: (1) biochar, purchased from Jinsheng Activated Carbon Co., Ltd.; (2) calcium–magnesium phosphate fertilizer, manufactured by Hubei Jinmingzhu Chemical Co., Ltd., with a composition of ≥18.0% P_2_O_5_, ≥20% available Ca, ≥6.8% available Mg, and ≥20% soluble SiO_2_; and (3) limestone powder, obtained from a local market, with CaCO_3_ as its primary component.

### 2.3 Experimental design

The experiment was conducted from November 2023 to August 2025 under a rice-rapeseed rotation system. Six treatments were established: control (CK); conventional fertilization (F); straw return + conventional fertilization (FT); straw return + calcium–magnesium phosphate fertilizer + conventional fertilization (FGT); straw return + limestone powder + conventional fertilization (FST), and straw return + biochar + conventional fertilization (FBT). Each treatment was replicated three times, resulting in a total of 18 experimental plots arranged in a randomized block design. Each plot covered an area of 20 m^2^.

Before transplanting rapeseed and rice, the straw from the preceding crop was mechanically chopped and evenly distributed onto the soil surface. Fertilizers and amendments were then applied simultaneously, followed by manual incorporation into the 0–20 cm soil layer to ensure thorough mixing. Throughout the entire growth period, all plots received identical field management practices except for the specific materials applied according to the experimental treatments. The application rates of amendments and the amounts of returned straw for each treatment during the rice and rapeseed seasons are presented in Tables 1, 2, respectively.

**Table 1:** Specific application rate of each treatment in rice season (kg/hm^2^).

Treatment	Straw return amount	Calcium–magnesium phosphate fertilizer	Limestone powder	Biochar	N	P_2_O_5_	K_2_O
CK	—	—	—	—	—	—	—
F	—	—	—	—	180	90	75
FT	2,250	—	—	—	180	90	75
FGT	2,250	449.5	—	—	180	—	75
FST	2,250	—	7,500	—	180	90	75
FBT	2,250	—	—	7,500	180	90	75

“—” indicates that no relevant data is available in this article. CK denotes the control; F denotes conventional fertilization; FT denotes straw return + conventional fertilization; FGT denotes straw return + calcium–magnesium phosphate fertilizer + conventional fertilization; FST denotes straw return + limestone powder + conventional fertilization; and FBT denotes straw return + biochar + conventional fertilization.

**Table 2:** Specific application rate of each treatment in rapeseed season (kg/hm^2^).

Treatment	Straw return amount	Calcium–magnesium phosphate fertilizer	Limestone powder	Biochar	N	P_2_O_5_	K_2_O
CK	—	—	—	—	—	—	—
F	—	—	—	—	99	36	45
FT	6,000	—	—	—	99	36	45
FGT	6,000	199.5	—	—	99	—	45
FST	6,000	—	7,500	—	99	36	45
FBT	6,000	—	—	7,500	99	36	45

“—” indicates that no relevant data is available in this article. CK denotes the control; F denotes conventional fertilization; FT denotes straw return + conventional fertilization; FGT denotes straw return + calcium–magnesium phosphate fertilizer + conventional fertilization; FST denotes straw return + limestone powder + conventional fertilization; and FBT denotes straw return + biochar + conventional fertilization.

### 2.4 Soil sample collection and analysis

Soil samples were collected in late August 2025 after rice harvesting. For each plot, five soil samples were randomly collected from the 0–20 cm topsoil layer and thoroughly mixed to obtain a composite sample. Approximately 4 kg of soil was collected from each plot and transported to the laboratory in an insulated container with ice packs. After removing visible impurities, including stones and residual plant roots, each soil sample was evenly divided into three portions. One portion was immediately stored at −80 °C for soil microbial community analysis, another was stored at 4 °C for soil enzyme activity determination, and the remaining portion was air-dried, gently ground, and sieved through 2 mm and 0.149 mm meshes for soil pH and SOC analyses, respectively.

Soil pH was determined using a potentiometric method with a soil-to-water ratio of 1:2.5; SOC content was measured using the external heating potassium dichromate oxidation method (Lu, 2000).

Soil enzyme activity determination: Four soil enzymes, including soil urease (S-UE; G0301F), soil peroxidase (S-POD; G0317F), soil acid protease (S-ACPT; G0316F), and soil β-glucosidase (S-GC; G0312F48), were determined using commercial assay kits supplied by Suzhou Grace Biotechnology Co., Ltd. (Suzhou, China), following the manufacturer’s protocols. For S-UE, 0.10 g fresh soil was incubated at 37 °C for 24 h, and absorbance was measured at 578 nm after color development. For S-POD, 0.10 g fresh soil was incubated at 25 °C for 1 h, followed by absorbance measurement at 475 nm. For S-ACPT, 0.20 g fresh soil was incubated at 50 °C for 2 h, followed by color development at room temperature for 20 min and absorbance measurement at 680 nm. For S-GC, 0.05 g fresh soil was incubated at 37 °C for 1 h, and absorbance was measured at 405 nm. The standard curves for S-UE, S-ACPT, and S-GC were *y* = 1.0438x − 0.0015 (*R*^2^ = 0.9999), *y* = 0.1144x + 0.0034 (*R*^2^ = 0.9994), and y = 0.1461x + 0.0087 (*R*^2^ = 0.9996), respectively. S-POD activity was calculated using the molar extinction coefficient specified in the kit manual. Enzyme activities were calculated according to the corresponding equations provided by the manufacturer and expressed according to the activity definitions of the respective assay kits. Because the formulations and concentrations of the proprietary reagents were not disclosed by the manufacturer, the corresponding kit numbers and key assay conditions are provided to facilitate methodological verification and reproducibility. Enzyme activities were calculated according to the equations and activity definitions provided in the respective assay kit manuals. S-UE activity was expressed as μg/d/g soil, S-POD activity as nmol/h/g soil, S-ACPT activity as μg/h/g soil, and S-GC activity as nmol/h/g soil (Ma et al., 2025).

Soil bacterial community analysis: Total microbial genomic DNA was extracted from soil samples using the E.Z.N.A.® Soil DNA Kit according to the manufacturer’s protocol. The quality and integrity of extracted DNA were evaluated by 1.0% agarose gel electrophoresis, and DNA concentration and purity were determined using a NanoDrop 2000 spectrophotometer. The bacterial 16S rRNA gene was amplified using the universal primers 338F (5′-ACTCCTACGGAGGCAGCAG-3′) and 806R (5′-GGACTACHVGGGTWTCTAAT-3′). The purified PCR products were used to construct sequencing libraries and subjected to high-throughput sequencing. The obtained amplicon sequences were processed to generate amplicon sequence variants (ASVs). Representative ASV sequences were taxonomically annotated against a bacterial reference database. Subsequently, bacterial community composition, diversity indices (including Chao richness and Shannon diversity indices), and differentially abundant taxa were analyzed following established bioinformatic procedures (Zhou et al., 2024).

DNA extraction, PCR amplification, library construction, sequencing, and downstream data processing were performed by Majorbio Bio-Pharm Technology Co., Ltd. in Shanghai, China (Zhou et al., 2025).

### 2.5 Data processing and analysis

Raw data were organized using Microsoft Excel 2016. Sequencing data were analyzed using the MajorBio online platform^1^. Statistical analyses of soil physicochemical properties and enzyme activities were performed using IBM SPSS Statistics 26.0. Before statistical analysis, data were assessed for normality and homogeneity of variances. Treatment effects were evaluated using one-way analysis of variance (ANOVA). When the assumption of homogeneity of variances was met, Tukey’s HSD test was used for *post hoc* multiple comparisons; otherwise, the Games–Howell test was applied. Statistical significance was set at *p* < 0.05. Bacterial community composition was evaluated based on Bray–Curtis dissimilarities. Principal coordinate analysis (PCoA) was performed to visualize differences in bacterial community composition among treatments, and permutational multivariate analysis of variance (PERMANOVA) was used to assess treatment-related variation in overall bacterial community composition. Redundancy analysis (RDA) was conducted to examine the relationships between bacterial community composition and measured soil environmental variables. Environmental variables were standardized before RDA to minimize the influence of differences in measurement scales. Spearman rank correlation analysis was used to assess the relationships between the relative abundances of dominant bacterial taxa and soil pH, SOC, and enzyme activities. These analyses were used to identify statistical associations between bacterial community variation and soil environmental variables and were not intended to establish direct causal relationships. PICRUSt2 was used to infer the functional potential of bacterial communities from the 16S rRNA gene amplicon data. The resulting functional profiles were interpreted as predicted functional potential rather than direct measurements of bacterial functional activity. Statistical analyses were conducted using IBM SPSS Statistics 26.0 and R 4.5.1, and graphical visualization was performed using Origin 2021.

## 3 Result

### 3.1 Soil pH and SOC content

Straw return combined with amendments increased soil pH and alleviated soil acidification in acidic purple soil (Figure 1). Across all treatments, soil pH and SOC contents ranged from 5.02 to 6.97 and 21.86 to 26.41 g/kg, respectively (Figure 1a). Compared with CK, the FST and FBT treatments increased soil pH by 1.79 and 0.35 units, respectively. Relative to the F treatment, FST and FBT further elevated soil pH, with increases of 1.95 and 0.51 units, respectively (*p* < 0.05). These results indicate that straw return combined with amendments effectively mitigated soil acidification, with limestone powder exhibiting a stronger effect than biochar and calcium–magnesium phosphate fertilizer.

**Figure 1:**
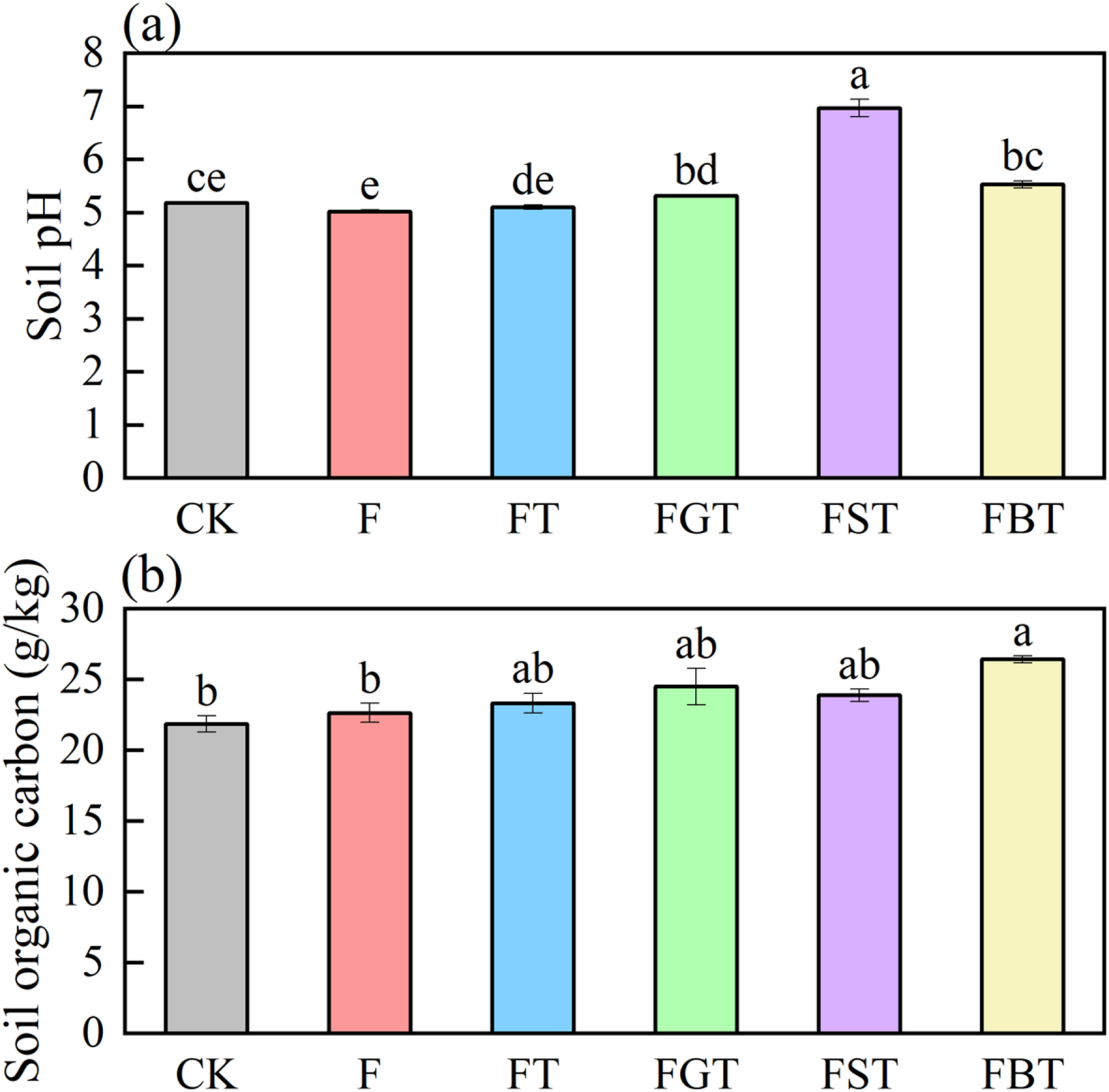
Effects of different treatments on soil pH and SOC. CK denotes the control; F denotes conventional fertilization; FT denotes straw return + conventional fertilization; FGT denotes straw return + calcium–magnesium phosphate fertilizer + conventional fertilization; FST denotes straw return + limestone powder + conventional fertilization; and FBT denotes straw return + biochar + conventional fertilization. Error bars represent the standard deviation of the mean (*n* = 3). Different lowercase letters indicate significant differences at the level of *p* < 0.05. **(a)** is soil pH, **(b)** is soil organic carbon (SOC).

Regarding SOC accumulation, compared with CK, the FST and FGT treatments increased SOC contents by 9.24 and 12.08%, respectively (Figure 1b). The FBT treatment resulted in the greatest increase in SOC content, significantly increasing SOC by 20.81 and 16.70% compared with CK and F, respectively (*p* < 0.05). This suggests that straw return combined with biochar not only improves the acid–base conditions of acidic purple soil but also promotes SOC accumulation by enhancing external organic carbon inputs, thereby promoting SOC accumulation.

Overall, different amendments exhibited distinct pathways for improving acidic purple soil properties. The primary advantage of FST treatment was its rapid neutralization of soil acidity, whereas FBT treatment mainly promoted SOC accumulation through biochar-derived carbon inputs.

### 3.2 Soil enzyme activities

Different treatments affected soil enzyme activities to varying degrees (Figure 2). Overall, straw return combined with amendments increased the activities of enzymes involved in carbon cycling and nitrogen transformation, with the FBT and FST treatments showing particularly pronounced activation effects. S-POD activity followed the order FST > FBT > FGT > F > FT > CK (Figure 2a), while S-UE activity showed the order FST > FBT > FGT > FT > F > CK (Figure 2b). S-ACPT and S-GC activity exhibited similar trends, with the orders of FBT > FGT > FST > FT > F > CK (Figures 2c,d), respectively. All amendment treatments resulted in higher enzyme activities than CK, indicating that external organic carbon inputs and regulation of soil acid–base conditions jointly influenced microbial-driven enzymatic processes.

**Figure 2:**
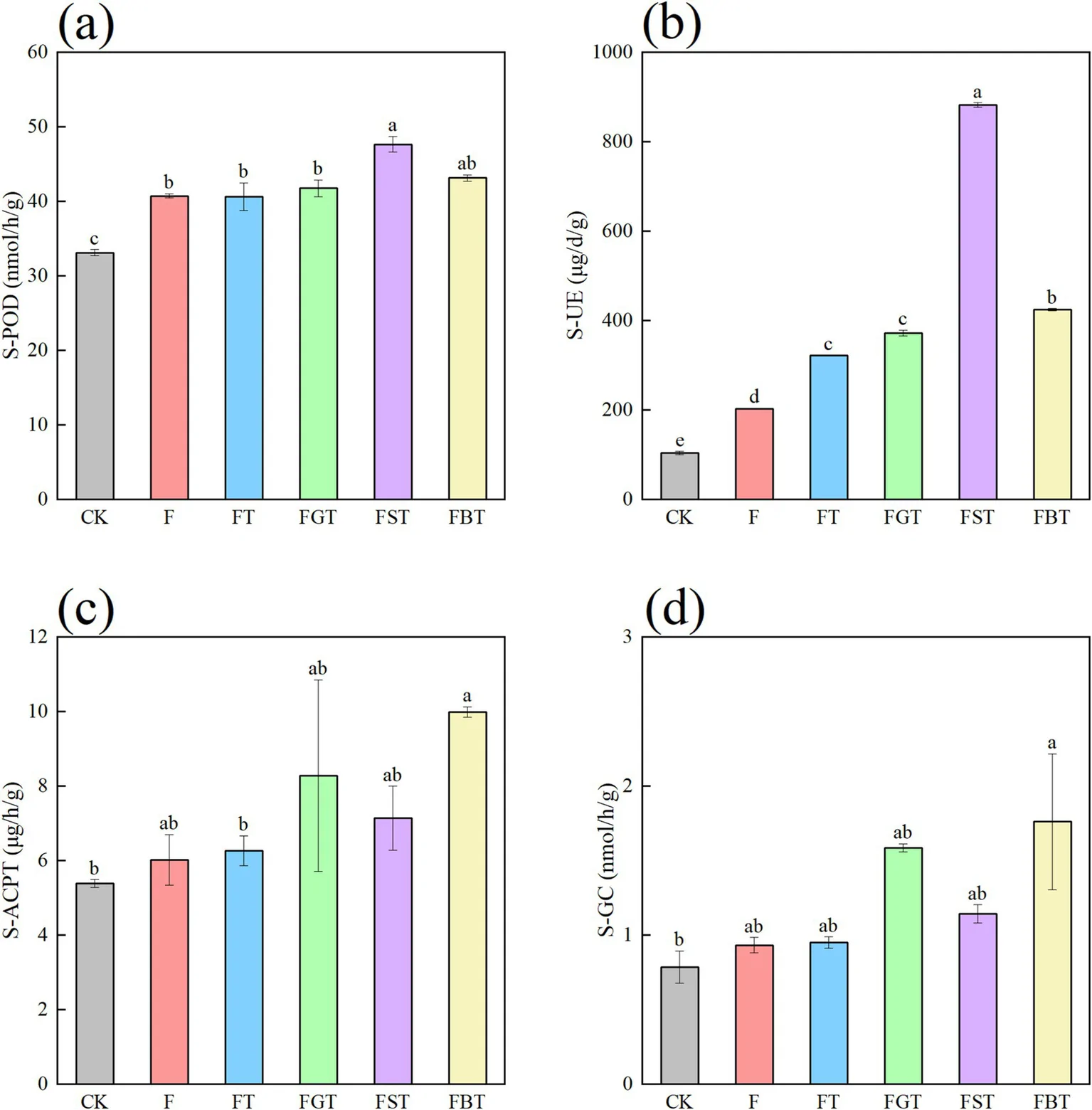
Effects of different treatments on soil enzyme activity. CK denotes the control; F denotes conventional fertilization; FT denotes straw return + conventional fertilization; FGT denotes straw return + calcium–magnesium phosphate fertilizer + conventional fertilization; FST denotes straw return + limestone powder + conventional fertilization; and FBT denotes straw return + biochar + conventional fertilization. Error bars represent the standard deviation of the mean (*n* = 3). Different lowercase letters indicate significant differences at the level of *p* < 0.05. **(a)** is soil peroxidase (S-POD), **(b)** is soil urease (S-UE), **(c)** is soil acid protease, **(d)** is soil β-glucosidase (S-GC).

Specifically, S-POD activity ranged from 33.13 to 47.67 nmol/h/g, with the highest value observed under the FST treatment, which significantly increased S-POD activity by 43.89% compared with CK (*p* < 0.05). No significant differences were observed among the FBT, FGT, F, and FT treatments, suggesting that straw return combined with limestone powder exerted a stronger stimulatory effect on S-POD activity. Compared with CK and F treatments, straw return combined with amendments induced greater responses in S-UE activity. The FST treatment exhibited significantly higher S-UE activity than other treatments (*p* < 0.05), suggesting that the increased soil pH under FST may have contributed to enhanced urease activity.

The activity of S-ACPT ranged from 5.38 to 9.99 μg/h/g, with the FBT treatment showing the greatest enhancement, increasing by 85.69% relative to CK (*p* < 0.05). The FT, FGT, and FST treatments also maintained relatively high S-ACPT activities. A similar pattern was observed for S-GC activity, which ranged from 0.79 to 1.76 nmol/h/g. The highest S-GC activity was detected under the FBT treatment, with a significant increase of 122.78% compared with CK (*p* < 0.05). In addition, the FGT treatment increased S-GC activity to 1.59 nmol/h/g, representing a 101.27% increase relative to CK, indicating a stimulatory effect on carbon degradation processes. In contrast, the F treatment showed relatively limited improvements in the activities of these enzymes, with overall lower levels compared with the amendment treatments.

Collectively, straw return combined with amendments enhanced the activities of key soil enzymes, with FBT and FST exhibiting the strongest stimulatory effects. The FST treatment primarily promoted enzyme activities involved in nitrogen transformation by improving soil acid–base conditions, whereas the FBT treatment enhanced carbon-degrading enzyme activities through additional carbon inputs and optimization of the soil microenvironment. These findings suggest that limestone powder and biochar regulate soil biogeochemical cycling through distinct functional pathways, highlighting the complementary roles of different amendments in improving soil ecological functions.

### 3.3 Soil bacterial community diversity analysis

Different treatments exerted varying effects on soil bacterial community richness, diversity, and ASV distribution patterns (Figure 3). The Venn diagram showed that a total of 447 shared ASVs were identified across all treatments, indicating that some bacterial ASVs were shared among different treatments (Figure 3a). Meanwhile, each treatment harbored a considerable number of unique ASVs. The FST treatment exhibited the highest number of unique ASVs (1171), followed by FGT (1108) and FBT (963), whereas CK showed the lowest number (877).

**Figure 3:**
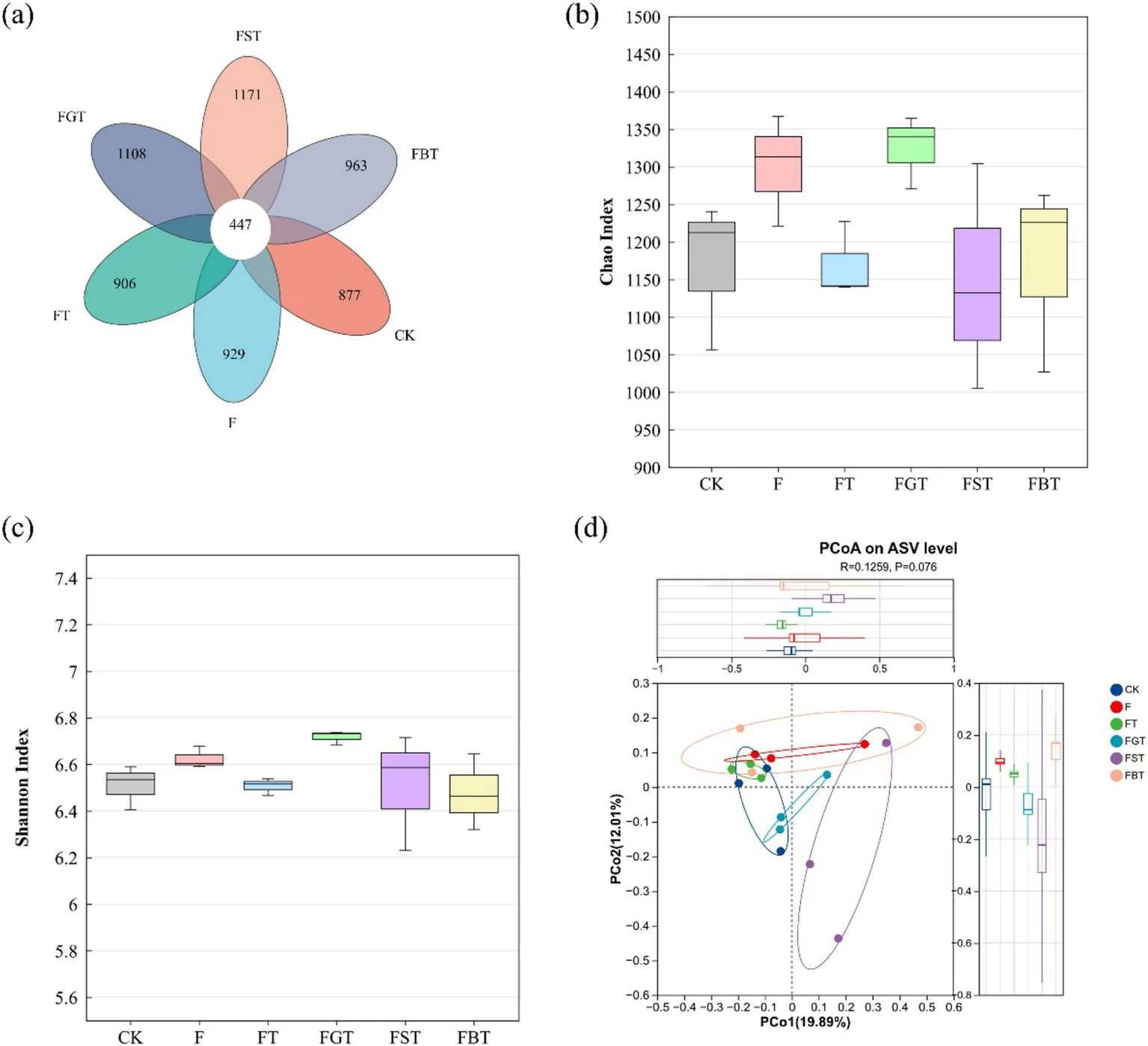
Effects of different treatments on soil bacterial community diversity. Panel **(a)** is Venn diagram, **(b)** is Chao index, **(c)** is Shannon index, **(d)** is PCoA based on bray-curtis distance. CK denotes the control; F denotes conventional fertilization; FT denotes straw return + conventional fertilization; FGT denotes straw return + calcium–magnesium phosphate fertilizer + conventional fertilization; FST denotes straw return + limestone powder + conventional fertilization; and FBT denotes straw return + biochar + conventional fertilization. Error bars represent the standard deviation of the mean (*n* = 3). Different lowercase letters indicate significant differences at the level of *p* < 0.05.

Alpha diversity analysis revealed that different treatments had effects on bacterial community richness and diversity (Figures 3b,c). The Chao index, representing bacterial community richness, showed that FGT and F treatments exhibited higher richness than CK. However, the Chao index of the FST treatment displayed relatively large variations among replicates, indicating lower stability of bacterial richness within this treatment. The Shannon index, which reflects community diversity and evenness, showed no significant differences among treatments. Notably, the FGT treatment maintained a relatively high Shannon index with low variation among replicates, suggesting that FGT tended to maintain a relatively balanced bacterial community. In contrast, the Shannon indices of FBT and FST treatments showed greater fluctuations, indicating that these amendments had stronger effects on bacterial community evenness. Overall, the amendment strategies primarily affected the abundance of specific bacterial taxa, while their impacts on overall community evenness were relatively limited.

PCoA further illustrated the effects of different treatments on soil bacterial community structure (Figure 3d). The first two principal coordinates explained 31.90% of the total variation in bacterial community composition, with PCo1 and PCo2 accounting for 19.89 and 12.01%, respectively. The ordination plot showed a certain degree of separation among treatments, although the overall treatment effect was not statistically significant according to PERMANOVA (*R*^2^ = 0.1259, *p* = 0.076). The FBT samples showed a relatively apparent separation trend from several other treatments, whereas the FST samples displayed greater dispersion along the PCo2 axis. In contrast, the F, FT, and FGT treatments showed relatively greater overlap in the ordination space. These patterns indicate variation in bacterial community composition among treatments, but the overall differences should be interpreted as trends rather than statistically significant treatment-induced shifts in community structure.

### 3.4 Composition of soil bacterial communities

At the phylum level, the bacterial communities across all treatments were primarily dominated by *Chloroflexota, Pseudomonadota, Actinomycetota, Bacillota*, and *Acidobacteriota*, which collectively accounted for more than 70% of the total bacterial relative abundance and represented the dominant bacterial phyla in acidic purple soil (Figure 4a). Compared with CK, all amendment treatments increased the relative abundance of *Chloroflexota*, which may be associated with increased organic carbon availability derived from straw decomposition. The relative abundance of *Actinomycetota* increased under FT and FBT treatments, likely due to the continuous input of lignocellulosic compounds from straw and biochar, which provided suitable substrates for *Actinomycetota* proliferation. In contrast, the abundance of *Actinomycetota* decreased under FGT and FST treatments, possibly due to the substantial increase in soil pH following the application of calcium–magnesium phosphate fertilizer and limestone powder. *Acidobacteriota*, a bacterial phylum commonly associated with acidic soils, exhibited relatively higher abundances under F, FT, and FST treatments. Meanwhile, *Bacillota* and *Thermodesulfobacteriota* were generally enriched in amendment treatments, suggesting their stronger adaptability to the modified soil environment.

**Figure 4:**
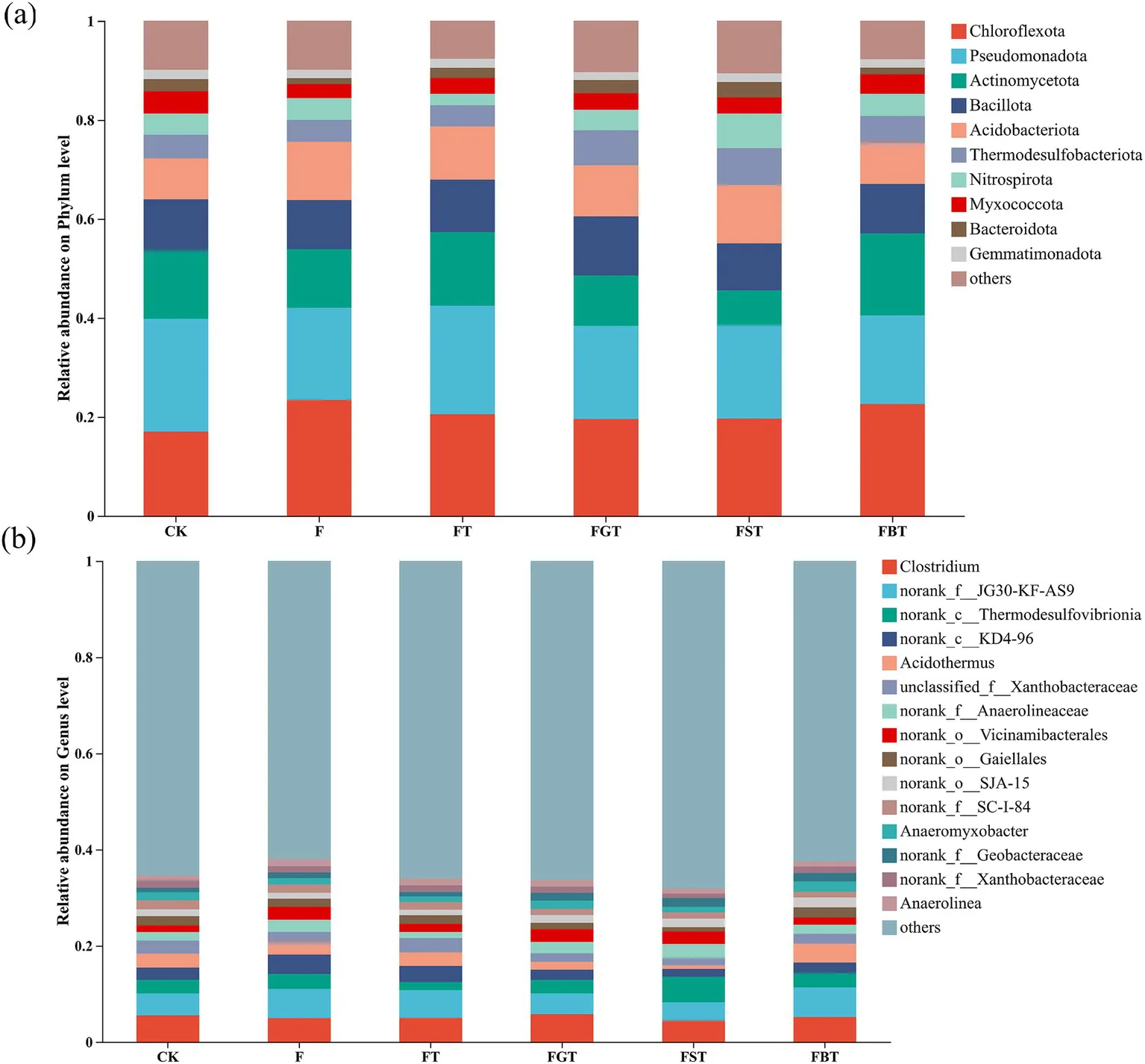
Effects of different treatments on the composition of soil bacterial communities. **(a)** Composition of the top 10 bacterial phyla; **(b)** composition of the top 15 bacterial genera. CK denotes the control; F denotes conventional fertilization; FT denotes straw return + conventional fertilization; FGT denotes straw return + calcium–magnesium phosphate fertilizer + conventional fertilization; FST denotes straw return + limestone powder + conventional fertilization; and FBT denotes straw return + biochar + conventional fertilization.

At the genus level, the dominant bacterial genera mainly included *Clostridium*, *Acidothermus*, *Anaeromyxobacter*, *Anaerolinea*, and a large proportion of unclassified genera (Figure 4b). Although the overall composition of dominant bacterial genera was similar among treatments, their relative abundances varied considerably. Compared with CK, the relative abundances of *Acidothermus* and *Anaerolinea* increased under F, FT, and FBT treatments, indicating their positive responses to chemical fertilization and straw-derived organic matter inputs. *Anaeromyxobacter* and *norank_f__Geobacteraceae*, which are involved in iron reduction processes and soil biogeochemical cycling, were also enriched in several amendment treatments. In addition, several unclassified taxa, including *norank_f__Xanthobacteraceae*, *norank_f__Vicinamibacterales*, and *norank_f__Gaiellales*, which are potentially associated with carbon and nitrogen cycling, exhibited increased abundances under certain amendment conditions. These results suggest that straw return combined with amendments was associated with differences in the relative abundance of potential functional taxa.

Overall, all treatments exerted varying degrees of influence on bacterial community composition, with FT, FST, and FBT treatments showing stronger effects on the relative abundance of several dominant bacterial taxa.

### 3.5 Multigroup comparison of bacterial communities among treatments and prediction of bacterial community functions

Multiple comparison analysis of dominant bacterial taxa revealed that the relative abundances of the 10 dominant bacterial taxa differed significantly among treatments (*p* < 0.05), indicating differences in the distribution of several dominant bacterial taxa among the amendment treatments (Figure 5a). Among these taxa, *norank_f__Bacteroidetes_vadinHA17* exhibited the highest abundance and was significantly enriched in the FGT and FST treatments. The nitrogen-fixing genus *Bradyrhizobium* showed the highest relative abundance under the FT treatment. In addition, *norank_f__PHOS-HE36* and *norank_f__Pedosphaeraceae* were significantly enriched in the FST treatment. Meanwhile, bacterial taxa such as *Syntrophorhabdus, Bauldia*, and *norank_o__CCD24* displayed pronounced variations among treatments, further suggesting that different amendment treatments were associated with differences in the relative abundances of specific bacterial taxa with potential ecological functions.

**Figure 5:**
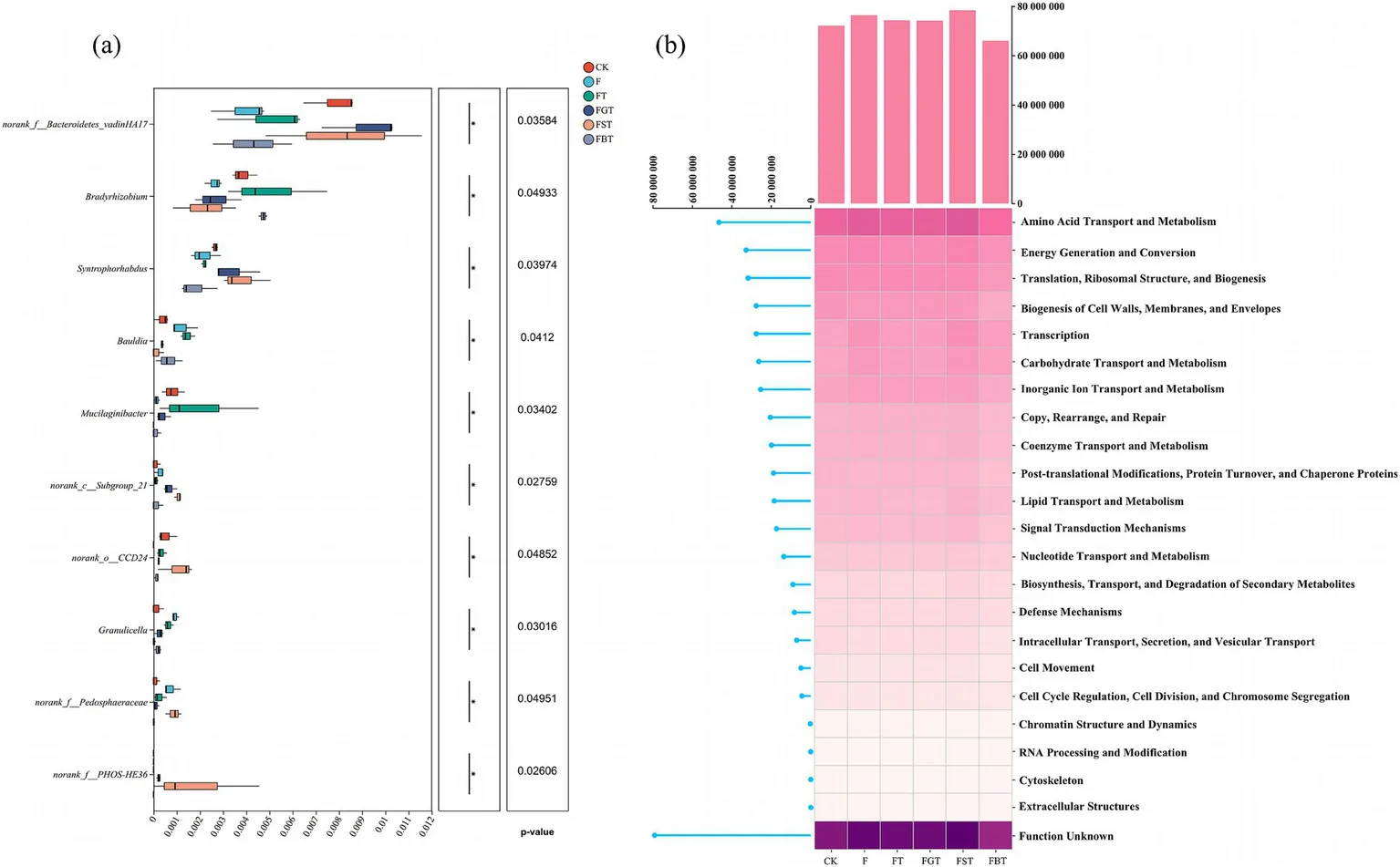
Differential analysis and predicted functional profiles of soil bacterial communities. **(a)** Multiple-group comparison of dominant bacterial taxa; **(b)** PICRUSt2-based prediction of bacterial functional potential. CK denotes the control; F denotes conventional fertilization; FT denotes straw return + conventional fertilization; FGT denotes straw return + calcium–magnesium phosphate fertilizer + conventional fertilization; FST denotes straw return + limestone powder + conventional fertilization; and FBT denotes straw return + biochar + conventional fertilization.

PICRUSt2 analysis showed that the predicted functional profiles of soil bacterial communities were broadly similar among treatments, with metabolism-related categories accounting for a large proportion of the predicted functions (Figure 5b). Differences in the predicted relative abundances of functional categories were observed among treatments, with FST showing relatively higher predicted abundances for several pathways than the other treatments. The major predicted functional categories included Amino acid transport and metabolism, Energy Generation and Conversion, Translation, Ribosomal Structure and Biogenesis, Biogenesis of Cell Walls, Membranes, Envelope, Transcription, and Carbohydrate Transport and Metabolism. These results indicate differences in the predicted functional potential of bacterial communities among treatments. However, because these functions were inferred from 16S rRNA gene profiles using PICRUSt2, they should be interpreted as predicted functional potential rather than direct measurements of bacterial metabolic activity or process rates in soil.

### 3.6 Relationships among soil bacterial communities, pH, SOC, and enzyme activities

The dominant bacterial genera exhibited distinct correlation patterns with soil pH, SOC, and enzyme activities (Figure 6a). Overall, *norank_o__SJA-15*, *norank_f__Geobacteraceae*, *norank_f__Anaerolineaceae*, *norank_o__Vicinamibacterales*, *Clostridium*, and *Anaeromyxobacter* showed generally positive correlations with soil pH, SOC, and enzyme activities. Among them, *norank_o__SJA-15* displayed significant or highly significant positive correlations with pH, SOC, UE, and GC, suggesting that these taxa were associated with soil environments characterized by higher pH and SOC levels. These associations indicate that these taxa were closely related to soil conditions characterized by higher pH and SOC levels. In contrast, *unclassified_f__Xanthobacteraceae*, *norank_f__SC-I-84*, and *norank_c__KD4-96* showed negative correlations with pH, SOC, and enzyme activities, with particularly strong negative associations with pH, UE, and GC. These negative associations suggest that the relative abundances of these taxa were higher under conditions characterized by lower pH and SOC and were inversely related to the measured enzyme activities. Other unclassified bacterial groups exhibited relatively weak relationships with environmental variables, indicating limited responses to changes in soil conditions. Collectively, higher soil pH and SOC levels, together with greater enzyme activities, were associated with differences in the relative abundances of several bacterial taxa.

**Figure 6:**
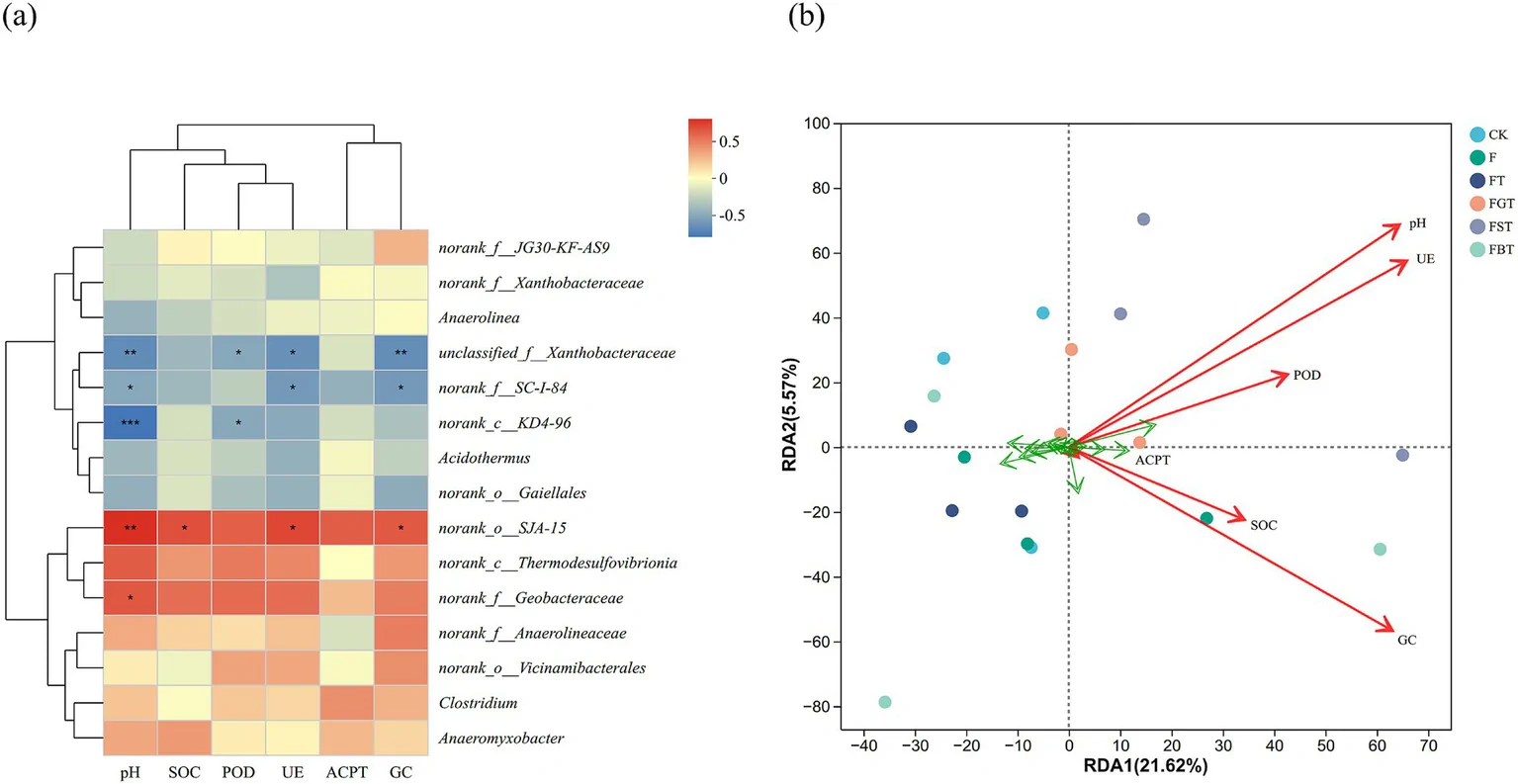
Relationship between soil bacterial communities and pH and SOC and enzyme activity. **(a)** Correlation heatmap with cluster dendrogram illustrating the correlations between soil bacterial genera and environmental variables (pH, SOC, POD, UE, ACPT, GC). **(b)** Redundancy analysis (RDA) ordination plot showing the relationships between soil bacterial community composition and environmental variables.

RDA revealed that the first two axes explained 27.37% of the variation in bacterial community composition at the genus level (Figure 6b). Among the environmental variables, pH and S-UE showed relatively strong associations with bacterial community variation. SOC, POD, and GC also showed considerable explanatory contributions, whereas ACPT had a relatively weaker influence. The observed bacterial community differentiation was associated with changes in soil acidity, organic carbon availability, and enzyme activities under the different amendment treatments. The green vectors represented the top 15 dominant bacterial genera, with vector direction and length reflecting their responses to soil environmental factors. Most dominant genera were located near the center of the ordination diagram, suggesting broad ecological adaptability and relatively stable responses to environmental fluctuations. Several genera were aligned with pH and S-UE vectors, indicating that their enrichment coincided with soil amelioration and enhanced nutrient cycling capacity. Conversely, a small number of bacterial taxa exhibited opposite orientations to environmental variables, suggesting their preference for acidic and nutrient-poor soil conditions.

Overall, variation in bacterial community composition among treatments was associated with differences in soil pH, SOC, and enzyme activities.

## 4 Discussion

### 4.1 Effects of straw return combined with amendments on soil pH and SOC

Straw return is an important agronomic practice for organic fertilization, which can effectively enhance soil fertility and ameliorate acidic soil conditions, and has been widely applied for improving cultivated land quality (Wu et al., 2026). Considering the limitations associated with the application of straw return or amendments alone for acidic soil remediation (Zhang et al., 2023), this study employed straw return as the foundation and introduced three types of amendments, including biochar, calcium–magnesium phosphate fertilizer, and limestone powder, to investigate their synergistic effects on the improvement of acidic purple soil. The results demonstrated that straw return combined with amendments increased soil pH and SOC content, effectively alleviating soil acidification while promoting organic carbon accumulation. Among all treatments, FST exhibited the most pronounced effect on increasing soil pH (Figure 1). Similarly, Liao et al. (2020) reported that the combined application of straw return and lime significantly alleviated soil acidification in acidic paddy soils. This improvement can be attributed to the abundant alkaline mineral elements, such as Ca, Mg, and K, contained in straw, which are gradually released during decomposition and mineralization, thereby reducing the accumulation of acidic substances and promoting the regulation of soil pH balance (Zhu et al., 2025). Meanwhile, limestone powder, primarily composed of calcium carbonate, possesses strong alkalinity. On the one hand, it can directly neutralize hydrogen ions and exchangeable aluminum ions in acidic soils, while the released Ca^2+^ can replace exchangeable Al^3+^ on soil colloids. On the other hand, it can neutralize organic acids generated during straw decomposition, thereby alleviating soil acidification through multiple synergistic mechanisms (Zeng et al., 2026). Although calcium–magnesium phosphate fertilizer also exhibits soil acidity-buffering capacity, its relatively low solubility results in a slower release of alkaline components and delayed pH regulation. In contrast, biochar mainly mitigates soil acidity through the adsorption of H^+^ by surface functional groups, and thus both amendments showed weaker effects than limestone powder (Zeng et al., 2026; Li et al., 2024b).

Unlike the pattern observed for soil pH, the FBT treatment showed superior performance in promoting soil organic carbon retention and accumulation. This was mainly because both straw and biochar are exogenous organic carbon inputs that directly supply substantial carbon sources to soils, thereby rapidly increasing SOC levels (Zong et al., 2025). In addition, biochar contains abundant stable aromatic carbon structures and possesses a unique porous architecture, which not only contributes to long-term carbon sequestration but also enhances the adsorption of dissolved organic carbon, reduces organic carbon mineralization, and minimizes carbon losses from soils (Tian et al., 2026). In contrast, limestone powder and calcium–magnesium phosphate fertilizer are inorganic amendments that cannot provide additional organic carbon inputs; therefore, their effects on SOC enhancement mainly rely on straw-derived carbon, resulting in relatively limited carbon accumulation. Consistently, Cong et al. (2020) demonstrated that both straw return and biochar application significantly increased soil organic carbon content.

Overall, the functional mechanisms of different treatments showed differentiation in acidic purple soil improvement. The primary contribution of FST was associated with the strong acid-neutralizing capacity of limestone powder and its ability to alleviate aluminum toxicity. The advantage of FBT mainly involved enhanced carbon input and stabilization mediated by biochar, thereby increasing soil carbon storage capacity. Meanwhile, FGT improved soil quality through the combined effects of moderate acidity regulation and the supplementation of Ca, Mg, and Si nutrients from calcium–magnesium phosphate fertilizer. These findings indicate that straw return combined with different amendments exerts distinct functional roles in regulating soil acidification and carbon sequestration processes in acidic purple soils.

### 4.2 Effect of straw return combined with amendments on soil enzyme activity

Soil enzymes are key biological catalysts driving soil organic matter decomposition, nutrient cycling, and energy transformation. Their activities provide direct insights into microbial metabolic capacity and soil biological fertility, while environmental factors such as soil pH, organic matter availability, and nutrient status are critical regulators of enzymatic processes (Liu C. et al., 2024; Liu Y. J. et al., 2024; Zheng et al., 2020). In this study, all amendment treatments increased soil enzyme activities compared with CK; however, different amendment strategies exhibited distinct functional preferences in regulating specific enzymes. Specifically, the FST treatment showed the greatest enhancement of S-POD and S-UE activities, whereas the FBT treatment exhibited a superior effect on increasing S-ACPT and S-GC activities (Figure 2). The two enzymes associated with N transformation, S-UE and S-ACPT, displayed distinct response patterns to amendment application. The highest increase in S-UE activity was observed under the FST treatment, which was closely associated with the ameliorating effect of limestone powder on acidic soils. As a key enzyme involved in urea hydrolysis and nitrogen transformation, S-UE activity is highly sensitive to soil pH conditions. Therefore, the higher S-UE activity observed under FST suggests an increased potential for urea-derived nitrogen transformation following soil acidification alleviation. However, because nitrogen mineralization rates and nitrogen fluxes were not directly measured in this study, the increase in S-UE activity should be interpreted as a change in the potential for nitrogen transformation rather than direct evidence of increased nitrogen mineralization. Li et al. (2010) reported that urease activity in grey brown purple soil reached its optimum under neutral pH conditions, whereas soil acidity significantly inhibited its enzymatic function. The soil used in this study was also classified as grey brown purple soil with pronounced acidification characteristics, which likely restricted S-UE activity under the initial acidic conditions. The application of limestone powder in the FST treatment could neutralize soil acidity through its buffering capacity, increase soil exchangeable calcium levels, and alleviate the inhibitory effects of low pH on S-UE activity (Zeng et al., 2026), thereby stimulating urease activity. Similarly, Wang et al. (2026) demonstrated that straw return combined with lime application improved soil acid–base conditions and nutrient availability, enhanced the rhizosphere environment, and continuously supplied carbon and nitrogen substrates for soil microorganisms, thereby alleviating microbial metabolic constraints and promoting soil enzyme activities. In contrast to S-UE, S-ACPT activity showed the strongest response under FBT. As an enzyme involved in protein decomposition, acid protease participates in the hydrolysis of proteinaceous organic matter and can contribute to the release of soluble organic nitrogen compounds during organic matter decomposition (Gao et al., 2025). The markedly higher S-ACPT activity under FBT therefore suggests an enhanced potential for the decomposition of organic nitrogen-containing substrates. This response may facilitate subsequent microbial nitrogen transformations by increasing the availability of soluble substrates, although the present study did not directly quantify nitrogen mineralization or the resulting nitrogen flux.

The significant increase in S-GC activity under the FBT treatment further demonstrated that straw return combined with biochar effectively enhanced soil carbon metabolic functions. On the one hand, straw return introduces abundant natural organic compounds, including cellulose, hemicellulose, and lignin, into soils, thereby increasing the availability of microbial substrates and stimulating microorganisms to produce more carbon-degrading enzymes, which accelerates soil organic carbon decomposition and transformation (Gai et al., 2025). On the other hand, biochar exerts multiple regulatory effects on soil enzyme dynamics. Its adsorption capacity enables the immobilization of extracellular enzymes, reduces enzymatic degradation by soil proteases, delays enzyme deactivation, and maintains long-term enzymatic stability (Gao et al., 2025; Zhu et al., 2017). Furthermore, biochar improves soil pore structure and enhances aeration and water permeability, creating a more favorable oxygen distribution environment for carbon-degrading microorganisms, such as aerobic bacteria and fungi. These improvements further stimulate the production and activity of enzymes associated with cellulose and lignin degradation (Gao et al., 2025). The marked increase in S-GC activity under FBT therefore indicates a greater potential for the decomposition of plant-derived carbohydrates and the release of soluble carbon substrates for microbial utilization. This process represents an important biochemical step in soil carbon transformation and may contribute to subsequent microbial carbon processing. Nevertheless, the present study measured SOC content and enzyme activity rather than carbon mineralization or other carbon fluxes; thus, the observed increase in S-GC activity reflects a change in carbon transformation potential rather than a quantified increase in carbon-cycling flux. The findings of this study are consistent with previous research. Su et al. (2024) reported that biochar application is an effective strategy for enhancing soil enzyme activities. Under appropriate application rates, biochar significantly increased the activities of various functional enzymes, including dehydrogenase, urease, and protease, thereby activating soil biological metabolism. Overall, the contrasting enzyme responses indicate that the three amendment strategies affected soil biochemical processes in different ways. FST mainly enhanced S-UE and S-POD under improved soil pH conditions, suggesting greater potential for urease-mediated nitrogen transformation and oxidative organic matter turnover. In comparison, FBT produced the greatest increases in SOC, S-GC, and S-ACPT, indicating enhanced potential for the decomposition and transformation of plant-derived carbon and organic nitrogen-containing substrates. FGT showed intermediate responses across the measured enzymes, suggesting a relatively moderate effect on soil biochemical processes. These results provide biochemical evidence for amendment-specific changes in nutrient transformation potential, but direct nutrient cycling fluxes were not quantified in this study.

### 4.3 Effect of straw return combined with amendments on bacterial community structure

Soil pH, SOC content, and nutrient availability are recognized as key environmental factors regulating soil bacterial community composition in agricultural ecosystems (Hu et al., 2024). Previous studies have demonstrated that agronomic practices, such as straw return combined with lime application, can substantially reshape soil bacterial community structures (Zhang et al., 2024). In the present study, differences in bacterial community composition were observed among treatments, although the overall treatment effect was not statistically significant according to PERMANOVA (*R*^2^ = 0.1259, *p* = 0.076). The greater numbers of unique ASVs observed under FST, FGT, and FBT provide evidence of variation in ASV composition among treatments, but these patterns should be interpreted as descriptive differences rather than statistically confirmed shifts in the overall bacterial community structure. Alpha-diversity analysis showed that FGT had a relatively high Chao richness and Shannon diversity, whereas the Shannon index did not differ significantly among treatments. Thus, the amendment treatments appeared to be associated with variation in bacterial richness and the relative abundance of specific taxa, while evidence for a significant change in overall community structure remained limited. PCoA analysis showed apparent differences in bacterial community composition among treatments, although the overall separation was not statistically supported by PERMANOVA. The FBT samples showed a relatively greater separation from the other treatments in the ordination space, whereas the FST samples showed relatively greater dispersion. These variations were likely associated with the distinct physicochemical properties of different amendments. Biochar and limestone powder can alleviate soil acidification, increase SOC accumulation, and improve microbial nutrient availability, thereby creating more favorable habitats for microbial colonization and growth (Gao et al., 2025; Hu et al., 2025). Low soil pH in acidic soils generally restricts microbial growth and proliferation, whereas the addition of external organic carbon provides abundant substrates for microbial metabolism, ultimately contributing to differences in bacterial community composition and structure (Zeng et al., 2026). The lack of a statistically significant overall treatment effect may be related to the relatively high background variability of soil bacterial communities and the substantial overlap among treatments. Although the amendment treatments altered soil pH, SOC, and enzyme activities, these changes may not have been sufficiently large or consistent among replicates to produce a statistically detectable shift in the overall bacterial community within the two-year experimental period. In addition, soil bacterial communities can be influenced by multiple interacting factors, including seasonal conditions, crop growth, nutrient availability, and microsite heterogeneity, which may have contributed to variation among replicates and reduced the apparent treatment effect. Thus, the separation observed in the PCoA should be considered a potential response pattern associated with the amendment treatments rather than evidence of significant restructuring of the overall bacterial community.

The present study demonstrated that *Chloroflexota, Pseudomonadota, Actinomycetota, Bacillota,* and *Acidobacteriota* were the dominant bacterial phyla across all treatments, which was consistent with previous studies by Liu C. et al. (2024), Liu Y. J. et al. (2024), and Wang et al. (2026) Although *Acidobacteriota* is generally considered an acidophilic bacterial group, different subgroups within this phylum exhibit considerable variation in their pH adaptability. Some subgroups preferentially inhabit strongly acidic environments, whereas others can survive under weakly acidic to neutral conditions. In the present study, chemical fertilization alone (F treatment) maintained strongly acidic soil conditions, resulting in the enrichment of acid-adapted *Acidobacteriota* subgroups. In the FT treatment, straw return provided oligotrophic carbon substrates, which supported the growth and proliferation of *Acidobacteriota*. Interestingly, despite exhibiting the highest soil pH among all treatments, the FST treatment still maintained a relatively high abundance of *Acidobacteriota*. This may be explained by the fact that limestone powder not only increased soil pH but also reduced aluminum toxicity and enhanced exchangeable calcium availability, thereby creating suitable conditions for the proliferation of specific *Acidobacteriota* subgroups. *Pseudomonadota* are highly sensitive to changes in soil labile carbon availability and are typical copiotrophic microorganisms. The input of external organic materials provides sufficient carbon substrates to support their growth and metabolic activities, while members of this phylum also participate actively in soil nitrogen transformation processes (Hilaire et al., 2026). *Actinomycetota* are widely distributed in soils and include taxa capable of utilizing or transforming relatively recalcitrant organic substrates, including lignocellulose-derived compounds. Such activities can contribute to the decomposition of plant residues and the turnover of complex organic matter. In the present study, the relative abundance of *Actinomycetota* increased under the FBT treatment. This response may be related to the combined effects of straw-derived organic substrates and the physicochemical changes associated with biochar addition. Straw return supplied lignocellulosic materials that could provide substrates for microorganisms involved in plant-residue decomposition, whereas biochar may have modified the soil carbon environment and provided additional microsites for microbial colonization (Meng et al., 2024). However, because lignocellulose decomposition and microbial functional activity were not directly measured, the increase in *Actinomycetota* should be interpreted as an association with the altered soil environment rather than direct evidence of enhanced lignocellulose decomposition. *Chloroflexota* can utilize intermediate metabolites produced during straw decomposition as growth substrates, and their relative abundance increased under all amendment treatments compared with CK. *Bacteroidota* are considered key decomposers involved in soil organic nitrogen degradation. Their enrichment under the FGT, FST, and FBT treatments was consistent with the observed increases in soil urease and acid protease activities, suggesting enhanced nitrogen transformation potential. *Nitrospirota* exhibited higher relative abundance under the FST treatment, likely because limestone powder improved soil pH conditions and created a more favorable environment for nitrifying microorganisms, thereby promoting efficient soil nitrogen cycling (Cui et al., 2024). *Bacillota* possess strong stress tolerance and can effectively survive under acidic and nutrient-limited soil conditions.

Multiple comparison analysis at the genus level further indicated that different amendment types selectively enriched distinct functional bacterial taxa. For example, *norank_f__Bacteroidetes_vadinHA17* was strongly enriched under the FGT and FST treatments, suggesting its preference for calcium-rich and near-neutral soil environments. In contrast, the nitrogen-fixing genus *Bradyrhizobium* exhibited a stronger response under the FT treatment, indicating that soils without mineral amendment inputs may provide more suitable conditions for the proliferation of nitrogen-fixing bacteria. PICRUSt2 analysis indicated that the predicted functional profiles of bacterial communities were broadly similar among treatments, with metabolism-related categories representing a major proportion of the predicted functions. The FST treatment showed relatively higher predicted abundances for several functional categories, including Amino acid transport and metabolism, Energy Generation and Conversion, and Carbohydrate Transport and Metabolism. These patterns may reflect differences in the functional potential inferred from bacterial community composition under the different soil conditions. However, PICRUSt2 predictions are derived from 16S rRNA gene sequencing data and provide an inference of potential functional capacity rather than direct measurements of gene abundance, gene expression, metabolic activity, or biogeochemical process rates. Therefore, the differences observed in Figure 5b should be regarded as differences in predicted functional profiles rather than evidence of enhanced or reduced metabolic activity in the soil. Further studies using metagenomic or metatranscriptomic approaches together with targeted functional assays are needed to verify whether the predicted functional differences correspond to changes in actual bacterial activities under different amendment treatments.

Overall, the three amendment strategies were associated with distinct patterns of soil biochemical responses and bacterial community variation (Figure 7). The FBT treatment promoted the enrichment of heterotrophic microorganisms through the input of external organic carbon, whereas the FGT treatment maintained bacterial community stability through the gradual nutrient-release characteristics of calcium–magnesium phosphate fertilizer. In contrast, the FST treatment induced differences in bacterial community composition by substantially modifying soil pH. Soil pH and SOC availability were closely associated with bacterial community differentiation, as increased carbon availability and alleviation of soil acidity promoted the proliferation of dominant bacterial groups and ultimately altered bacterial community composition (Philippot et al., 2013). Redundancy analysis and correlation analysis further demonstrated that soil pH, SOC, and enzyme activities were closely associated with the observed variation in bacterial community composition. The correlation analysis showed that several dominant bacterial taxa exhibited significant positive or negative associations with these environmental variables, while the RDA further indicated that pH and S-UE were among the variables most strongly related to community variation. These results suggest that changes in soil acidity, carbon availability, and enzyme activities may be linked to the differences in bacterial community composition observed among treatments. However, because the correlation and ordination analyses describe statistical associations, they do not provide direct evidence that these environmental variables causally drove the observed bacterial community responses. Therefore, the relationships identified here should be interpreted as potential ecological associations rather than confirmed causal mechanisms.

**Figure 7:**
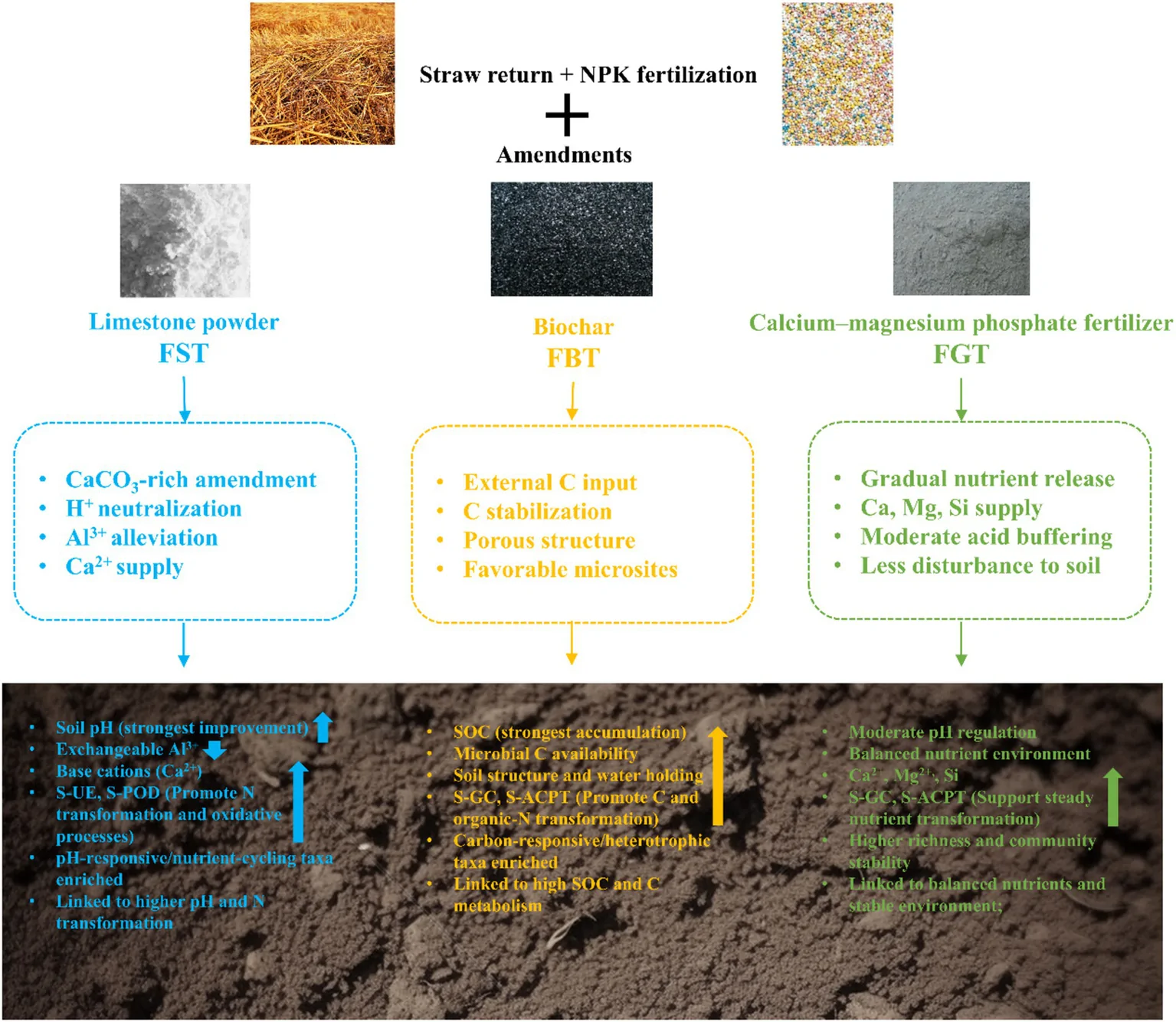
Mechanisms by which different amendment measures improve acidic purple soil.

Despite these findings, several limitations should be acknowledged. First, this study was conducted at a single experimental site, and the results may have been influenced by regional climatic conditions, inherent soil properties, and field management practices. Therefore, multi-site experiments are needed to evaluate the broader applicability of these findings. Second, bacterial community structures and potential functions were characterized solely based on 16S rRNA gene sequencing and PICRUSt2 prediction, which cannot fully represent microbial gene expression patterns and actual metabolic activities in soil. Moreover, this study focused exclusively on bacterial communities and did not consider interactions among fungi, archaea, soil fauna, and other biological components, nor did it systematically evaluate the long-term impacts of different amendments on soil carbon, nitrogen, and phosphorus cycling. Future research should therefore employ long-term field experiments to investigate the effects of straw return combined with different amendments on carbon sequestration, nutrient cycling, and microbial community stability in acidic purple soils. Integrating metagenomics, metatranscriptomics, and metabolomics approaches will further help identify metabolic regulatory pathways of key functional microorganisms and elucidate the molecular mechanisms underlying pH- and SOC-mediated microbial community reconstruction and restoration of soil ecological functions.

## 5 Conclusion

Straw return combined with different amendments generated distinct soil biochemical responses in acidic purple soil. FST was most effective in increasing soil pH and enhancing S-POD and S-UE activities, indicating its greater potential for alleviating soil acidity and improving soil enzymatic activity. FBT produced the greatest increase in SOC and the highest S-GC and S-ACPT activities, highlighting its potential for enhancing soil carbon accumulation and associated biochemical processes. FGT maintained relatively high bacterial diversity, suggesting a potential advantage for maintaining bacterial community diversity. Importantly, PERMANOVA did not detect a significant overall treatment effect on bacterial community structure (*R*^2^ = 0.1259, *p* = 0.076), although differences among treatments were observed in bacterial diversity and the relative abundance of several dominant taxa. Changes in bacterial communities were associated with soil pH, SOC, and enzyme activities, with pH and S-UE showing relatively strong associations in RDA. These results indicate potential links between amendment-induced changes in soil properties and bacterial community responses, but do not establish direct causal relationships. Under the conditions of this two-year field experiment, the three amendment strategies therefore appear to serve different management purposes: FST is preferable for rapid soil pH correction, FBT for increasing SOC and promoting carbon accumulation, and FGT for maintaining bacterial diversity while providing mineral nutrients. These treatment-specific benefits provide a basis for selecting amendments according to targeted management objectives in acidic purple soil of the Sichuan Basin.

## Data Availability

The original contributions presented in the study are included in the article/supplementary material, further inquiries can be directed to the corresponding author.

## Footnotes

1
https://www.majorbio.com
